# Distinct different expression of Th17 and Th9 cells in coxsackie virus B3-induced mice viral myocarditis

**DOI:** 10.1186/1743-422X-8-267

**Published:** 2011-06-02

**Authors:** Kong Qing, Wu Weifeng, Yang Fan, Yan Yuluan, Pang Yu, Huang Yanlan

**Affiliations:** 1 Guangxi Cardiovascular Institute, Shuang-Yong Road 6, Nanning, China; 2 Department of Cardiology, the First Affiliated Hospital of Guangxi Medical University, Shuang-Yong Road 6, 530021 Nanning, China

## Abstract

**Background:**

Recently, a new subset of CD4^+^T helper(Th) cell that predominantly secret cytokine interleukin-9(IL-9) is identified, termed Th9 cell. It has been reported to participate in tissue inflammation and autoimmune responses, and induce disease which differed from Th17 cells. Th17 cells have been shown to play a critical role in viral myocarditis (VMC), but whether Th9 cells are involved in the pathogenesis of VMC remains unclear.

**Results:**

BALB/c mice were intraperitoneally (i.p) injected with coxsackie virus B3(CVB3) for establishing VMC models. Control mice were treated with phosphate-buffered saline i.p. On day 0,7,14,21,28,35,42 after injection, myocardial histopathological changes were evaluated by hematoxylin-eosin staining. Splenic Th17 and Th9 cells subsets were analyzed by flow cytometry. And cardiac IL-17, IL-9 mRNA were measured by semi-quantitative reverse transcription-PCR and nested PCR, respectively. Results showed the levels of Th17 cells and IL-17 mRNA obviously increased in VMC mice on 7 day after infection, peaked on day 28, and highly persisted to at least day 42 (p < 0.05). While the frequencies of Th9 cells and IL-9 mRNA showed no significant difference between VMC and control group throughout the course of the experiment(p > 0.05).

**Conclusions:**

It was differentiated Th17 but not Th9 cells significantly elevated in the development of CVB3-induced VMC. The microenvironment of VMC seemed to contribute to the differentiation and proliferation of Th17 rather than Th9 cells. Our preliminary data implied Th9 cells could not protect against VMC nor promote the disease.

## Background

Viral myocarditis(VMC) is an important cause of heart failure in young adults and often progresses to chronic myocarditis, dilated cardiomyopathy(DCM) requiring heart transplantation[1]. CVB3, an enterovirus of Picornaviridae family, is believed to be the primary pathogen in human VMC[1,2]. Multiple studies had established that intraperitoneal inoculation of BALB/c mice with heart-passaged CVB3 (Nancy strain), can induce inflammatory heart disease which closely resembled human VMC[3], and thus this model has been widely used for investigating VMC. Previous experiments had indicated the major mechanisms in the pathogenesis of VMC were inflammation and autoimmune responses triggered by the viral infection and mediated by T cells[4,5]. Besides Th1,Th2 and Treg cells, our previous report and other researches demonstrated Th17 cells which preferentially secret cytokine IL-17 might play an important role in mediating inflammation of VMC[6-9]. But the fundamental mechanisms responsible for VMC are not completely clarified.

Recently, a novel independent Th-cell subset, "Th9" cells had been recognized[10,11]. Driven by the combined effects of TGF-β and IL-4, it produces large quantities of IL-9 which has long been thought to be a Th2 cytokine. Beside IL-10, Th9 cells secret small amounts of other Th2-related cytokines such as IL-4, IL-5 and IL-13. Although the mechanism for the differentiation and proliferation of Th9 cells have not been fully elucidated, the specific transcription factor PU.1 and interferon-regulatory factor 4 strongly support the existence and functional relevance of Th9 cells in vivo[12,13]. Developing literature has demonstrated that Th9 cells were associated with colitis, peripheral neuritis, experimental autoimmune encephalomyelitis (EAE) and allergic disease[11-17]. Especially, a recent study indicated that IL-9 involved in antiviral immunity, regulating T and B cell responses to respiratory syncytial virus infection in BALB/c mice[18]. It seems Th9 cells provide a unique contribution to tissue inflammation and immune responses, and participate in Th17-mediated diseases. Additionally, Anneli Jäger and colleagues reported Th17 and Th9 effector cells induced EAE, an animal model of human multiple sclerosis, with different pathological phenotypes, indicated distinct effector function of Th17 and Th9 cells[14]. Th17 cells had been shown to be associated to inflammation of CVB3-induced mice VMC, whether the unique IL-9 producing cells, Th9 participate in development of VMC and whether the expression level of Th17 differ from Th9 cells remain to be determined. In this study, we compared the Th9 and Th17 functions on different level including cell frequencies and major cytokine secretion to preliminarily explore whether Th9 cells involved in the pathogenesis of CVB3-induced mice VMC, and whether Th9 and Th17 cells differentially expressed in VMC.

## Methods

### Animals

Specific pathogen-free male BALB/c mice(6 weeks of age) were obtained from Shanghai Laboratory Animal Center, Chinese Academy of Sciences, Shanghai, China (Certificate No.0062353 -SCXK (SH) 2007-0005). All animals were housed under pathogen-free conditions at the Experimental Animal Center of the Guangxi Medical University. All experiments using mice were performed in accordance with protocols approved by Guangxi Medical University Animal Ethics Committee.

### Interventions and groups

Heart-passaged CVB3 (Nancy strain, from Institute of immunology of Guangxi Medical University) was maintained by passage through Hep-2 cells. 50% tissue culture infectious dose (TCID50) assay of Hep-2 cell was 1 × 10^-7^. The CVB3 was diluted in PBS (Solarbio Science & Technology Co, Ltd, Beijing, China). A total of 105 mice were randomly divided into VMC(n = 70,10 mice/subgroup) and control groups (n = 35,5 mice/subgroup). Each group was divided into 7 subgroups (Day0, Day7, Day14,Day21,Day28,Day35,Day42). The VMC group was treated with (i.p.) 0.1 ml PBS containing approximately 100TCID50 of the virus. Mice injected (i.p.) with 0.1 ml PBS were taken as control. The day of injection was defined as day 0. Surviving mice of 7 subgroups were separately sacrificed by cervical dislocation on 7 different time points, which were day 0,7,14,21,28,35,42 after injection. Hearts and spleens were removed aseptically as fresh specimens to be measured.

### Histopathological examination

The hearts were fixed in 10% formalin, then embedded in paraffin. After sectioned along the entire length of the heart into 5-μm sections, and stained with H&E (hematoxylin and eosin), histopathological change was observed by using light microscopy(Nikon Eclipse E800 Microscope, Kawasaki, Kanagawa, Japan). Pathological scores were graded by two independent researchers in a blinded manner according to the following scoring system: 0, no inflammatory infiltrates; 1, small foci of inflammatory cells between myocytes or inflammatory cells surrounding individual myocytes; 2, larger foci of 100 inflammatory cells or involving at least 30 myocytes; 3, 10% of a myocardial cross-section involved; 4, 30% of a myocardial cross-section involved[19].

### Flow cytometric analysis of Th17 and Th9 cells

Splenic cells were gently dispersed through nylon mesh into a single-cell suspension, washed with RPMI 1640(Gibco, USA). Lymphocytes were obtained by density gradient centrifugation from cells with Ficoll Paque (Solarbio Science &Technology). These cells were suspended in RPMI 1640 medium with 10% FCS (Gibco, USA) and transferred to each well of 24-well plates. Cultures were stimulated with phorbol myristate acetate (PMA, 25 ng/ml, Sigma-Aldrich, USA) and ionomycin (1 μg/ml, Sigma-Aldrich) in the presence of GolgiPlug (10^6^1 ul/cells, BD Biosciences) at 37°C under a 5% CO_2 _environment. After 4 h of incubation, cells were harvested and washed once with PBS. Then cells were incubated with phycoerythrin Cye-5-conjugated anti-mouse CD4 (PE-Cye-CD4, BD Biosciences). After washed with PBS, cells were fixed in 4% paraformaldehyde, permeabilized with 0.1% saponin. Then cells were stained with phycoerythrin-conjugated anti-mouse IL-17 and Alexa Fluor^® ^647 anti-mouse IL-9, and analyzed on a FACSCalibur flow cytometer (BD Bioscience). CellQuest software (BD Biosciences) was used for data acquisition.

### Polymerase chain reaction(PCR) of IL-17 and IL-9 mRNA

Total RNA of heart tissues was extracted using TRIZOL Reagent^® ^(Invitrogen, USA), and then converted into cDNA using an Reverse Transcription kit (Ferma, USA) according to the manufacturer's protocol. The primers for IL-17A, IL-9 and the housekeeping gene β-actin were designed by Primer Premier 5.0 (Table 1). For IL-17A amplification semi-quantitative reverse transcription-PCR was performed under the following conditions: pre-heating at 94°C for 3 min, denaturing at 94°C for 30 second(s), annealing at 64.9°C for 30 s, and extension at 72°C for 60 s. The reaction repeated for 35 cycles followed by incubation at 72°C for 10 min. Products were separated using 2% agarose gel electrophoresis and visualized by 0.5 mg/ml ethidium bromide staining and ultraviolet transillumination. The resulting bands (349 bp) were measured using the Digital Gel Imaging Analyst (Nikon 990-Doc 1000, USA). Background density was subtracted from each band and the relative IL-17 mRNA expressions were normalized to the level of β-actin transcripts. For IL-9, reverse transcription-PCR shown its mRNA was undetectable in heart. Then we amplified with nested PCR which contains the first and second round PCR reaction(PCR1 and PCR2). Specific primers were used for PCR1 reaction. After 3 min at 72°C, 40 amplification cycles were used (94°C for 45 s, 56°C for 45 s, and 72°C for 1 min), then 72°C for 6 min. PCR1 products and specific primers were used for PCR2. The polymerase was activated by incubation at 95°C for 3 min prior to 40 amplification cycles (94°C for 30 s, 58°C for 30 s, and 72°C for 30 s), followed by 3 min at 72°C. After PCR2 products were separated by electrophoresis, the purpose gene were investigated in all samples. IL-9 frequency was determined via direct counting. The presence of 140 bp band indicated the expression of IL-9mRNA, while its absence confirmed no expression. Sterile water was also amplified with nested PCR as the negative controls. Products were sequenced by Sangon Biological Engineering Technology & Services Co., Ltd., (Shanghai, China), and blasted in the NCBI Blast bank. All samples were measured in triplicate.

**Table 1:** Sequences of primers for PCR

Molecule	Sequence (5' -3')	Length
IL-17	sense:5'GTCAATGCGGAGGGAAAG 3'	349 bp
[GenBank:16171]	antisense:5'CACGAAGCAGTTTGGGAC 3'	

β-actin	sense: 5'CCAGCCTTCCTTCTTGGGTAT 3'	102 bp
[GenBank:11461]	antisense: 5'TTGGCATAGAGGTCTTTACGG 3'	

IL-9	PCR1 sense: 5'GCATCAGAGACACCAATTACCT 3'	140 bp
[GenBank:16198]	PCR1 antisense: 5' TTAAGGAGGGGAGGTTTTGTA 3'	
	PCR2 sense: 5'AACTGATGATTGTACCACACCGTGC 3'	
	PCR2 antisense: 5' AGGACGGACACGTGATGTTCTTTAG 3'	

### Statistical analysis

Quantitative variables were expressed as mean±standard deviation (SD). One-way ANOVA was used for comparison among groups. Qualitative variables of IL-9 mRNA measured by nested-PCR were expressed as percentages, and differences in IL-9 mRNA distribution among groups were obtained using the Chi-Square Tests. Statistical analyses were performed with the use of SPSS 13.0, p < 0.05 was considered statistically significant.

## Results

### Development of VMC

**Figure 1:**
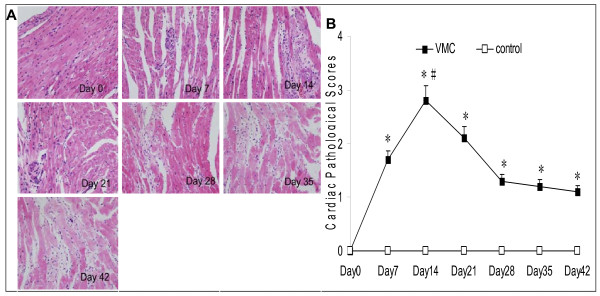
**The severity of VMC were evaluated by histopathological examination**. A. Representative of myocardial histopathologic images in VMC group(H&E, original magnification × 400). Myocardial was normal on Day 0. A few scattered small foci of myocyte necrosis were observed on Day 7. The severity of myocardial necrosis and interstitial inflammatory cell infiltration were markedly increased on Day 14. Then the degree of inflammation gradually decreased. Fibrous hyperplasia was found from Day 28 on. B. The results of statistical analysis for pathological scores in different groups. n = 5 mice for per control subgroup, n = 6-10 for per VMC subgroup. Values are expressed as mean ± SD. *, *p *< 0.01 vs. control subgroups sacrificed on the same time point; #, *p *< 0.05 vs. other VMC subgroups. VMC, Viral myocarditis.

Signs of VMC were apparent in the experimental group after virus injection, including weakness, coat ruffling, irritability, back arching, lethargy, anorexia, weight loss, and some mice died. Number of surviving mice in VMC subgroups from day 0 to 42 were: 10, 9, 7, 6, 6, 7 and 7, respectively. The cardiac pathological scores of VMC significantly increased on day 7 (1.8 ± 0.5), peaked on day 14 (2.8 ± 0.4). Then the severity of inflammation gradually ameliorated. In contrast, the control group showed no inflammatory cell infiltration, necrosis or fibrosis lesions in hearts, and no mice died(Figure 1).

### Distinct frequencies of Th17 and Th9 cells

**Figure 2:**
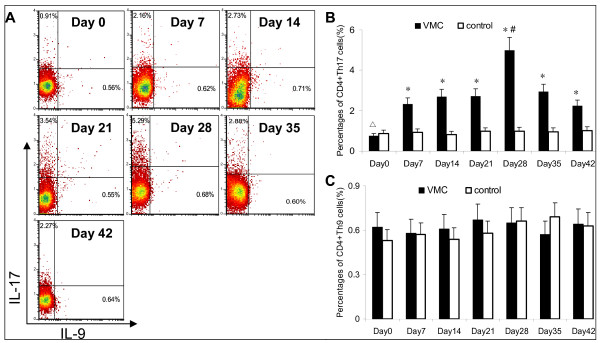
**The frequencies of Th17 and Th9 cells were investigated by flow cytometry**. A. Representative pictures for Th17 cells, and Th9 cells in VMC group. Numbers in upper left quadrants and lower right quadrants indicate the separate percentages of CD4^+^IL-17^+^IL-9^- ^Th17 cells and CD4^+^IL-9^+^IL-17^- ^Th9 cells, respectively. B,C. The results of statistical analysis for the alteration of Th17 and Th9 cells in different groups. n = 5 mice for per control subgroup, n = 6-10 for per VMC subgroup. Values are expressed as mean ± SD. *, p < 0.05 vs. control subgroups sacrificed on the same time point; #, p < 0.01 vs. other VMC subgroups; Δ, p < 0.01 vs. Day14,21,28,35,42 subgroups of VMC.

As shown in Figure 2, frequencies of splenic Th17 cells (CD4^+^IL17^+^/CD4^+^T cells) in VMC group from day 0 to 42 were: 0.76 ± 0.34%,2.32 ± 0.78%, 2.70 ± 0.86%, 2.72 ± 0.72%, 4.99 ± 0.85%, 2.94 ± 0.48%,2.24 ± 0.42%. Th17 cells in the control group from day 0 to 42 were 0.87 ± 0.45%, 0.93 ± 0.14%, 0.82 ± 0.09%, 0.97 ± 0.43%, 0.98 ± 0.42%, 0.96 ± 0.54%, 1.02 ± 0.63%. These data indicated the time-course of Th17 cells were significant higher than control group from day 7, and peaked on day 28, then wanning(p < 0.05). Frequencies of Th9 cells (CD4^+^IL9^+^/CD4^+^T cells) in VMC group from day 0 to 42 were: 0.62 ± 0.17%, 0.58 ± 0.21%, 0.61 ± 0.24%, 0.67 ± 0.28%,0.65 ± 0.25%,0.57 ± 0.28%,0.64 ± 0.26%. Th9 cells in the control group from day 0 to 42 were 0.53 ± 0.17%, 0.57 ± 0.23%, 0.54 ± 0.27%, 0.58 ± 0.16%, 0.66 ± 0.30%, 0.69 ± 0.32%,0.63 ± 0.27%. Results showed no significant difference between the VMC and control group on each time point(all p > 0.05).

### Different level of IL-17 and IL-9 mRNA

**Figure 3:**
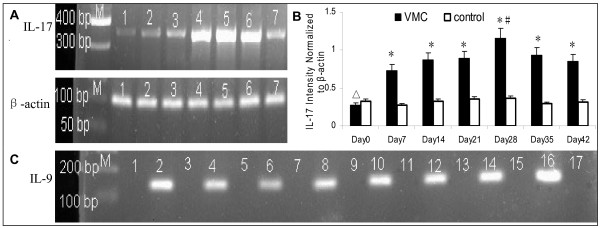
**The levels of cardiac IL-17 and IL-9 mRNA were detected by PCR**. A. Representative images showing semi-quantitative reverse transcription-PCR products for IL-17 mRNA transcription in VMC group. Lane M, 50-bp marker ladder;Lanes1-7, Day 0,7,14,21,28,35,42 subgroups. B.Collective analyses of results from all groups for IL-17 mRNA. The products were normalized versus β-actin mRNA band intensities. C. The percentages of cardiac IL-9 mRNA were detected by nested-PCR. Representative images showing PCR products of the samples. Lane M,100-bp marker ladder;Lanes1,2, samples of control group; Lanes 3-16, samples of different time point in VMC groups; Lanes 3,4, Day0; Lanes 5,6, Day7; Lanes 7,8, Day14; Lanes9,10, Day21; Lanes11,12, Day28; Lanes13,14, Day35; Lanes 15,16, Day42. Lane17, sterile water were amplified as the negative control. n = 5 mice for per control subgroup, n = 6-10 for per VMC subgroup. Values are expressed as mean ± SD. *, p < 0.01 vs. control subgroups sacrificed on the same point time; #, p < 0.05 vs. other VMC subgroups;Δ, p < 0.01 vs. Day14,21,28,35,42 subgroups of the VMC group.

As shown in Figure 3, for cardiac IL-17 mRNA, its intensity normalized to β-actin in VMC group from day 0 to 42 were: 0.27 ± 0.18, 0.73 ± 0.32, 0.87 ± 0.25, 0.89 ± 0.34, 1.16 ± 0.24, 0.93 ± 0.28, 0.85 ± 0.27. Data in the control group from day 0 to 42 were: 0.32 ± 0.11, 0.27 ± 0.15, 0.32 ± 0.18, 0.35 ± 0.13, 0.36 ± 0.17, 0.29 ± 0.14, 0.31 ± 0.18. In accordance with flow cytometry analysis of Th17 cells, IL-17 mRNA obviously upregulated in mice with VMC from day 7 and peaked on day 28(p < 0.05). For IL-9, the percentages of its cardiac mRNA in VMC group from day 0 to 42 were: 60%, 55.6%, 71.4%, 66.7%, 66.7%, 71.4%, 57.1%. Results in the control group were 60%, 40%, 60%, 60%, 60%,60% and 40%. Fisher's Exact Test was used to analyze the differences of IL-9 mRNA percentages between the VMC and control groups because of small size. No significant difference was observed throughout the course of the experiment(all p > 0.05), which was in agreement with our observations on the frequencies of Th9 cells.

## Discussion

The results of our experiment showed that the extent of myocardial inflammatory infiltrations peaked around day 14 after injection with CVB3. It was differentiated Th17 cell and its major cytokine IL-17 dramatically increased in VMC from day 7 post-infection, and persisted at least to day 42. But for Th9 cells frequencies, there was no obvious difference between VMC and control group throughout the course of the experiment. The similar phenomena also appeared on the level of IL-9 mRNA. In agreement with low frequencies of Th9 cells in spleens, we found cardiac IL-9 mRNA was undetectable by semi-quantitative reverse transcription PCR. Then we performed a more sensitive method for original specimens, nested PCR[20]. These manifestations suggested the Th9 cells in CVB3 and PBS treated BALB/c mice might be very low.

Several recent discoveries of Th9 cells found in mice and human have broadened our understanding of autoimmune disease, because this unique IL-9-producing subset seems can explain some phenomena underlying T cell-mediated tissue inflammation and antiviral immunities[21]. Th9 cells might contribute to immunity against EAE, colitis and peripheral neuritis, allergic inflammation, allergic rhinitis and Alzheimer's disease[11-17,22-24]. On the other hand, emerging literature identified IL-9 involved in anti-inflammatory, antiviral, nephroprotective activity, and enhanced the immunosuppressive function of Tregs[18,25,26]. These conflicting observations suggested Th9 cells can exert pro-inflammatory or anti-inflammatory activities, and might function as both a positive and negative regulator of immune response depending on the context. It is therefore possible that Th9 cells can protect against VMC or promote the disease, and may be differentially affected in mice with VMC or not, hence the present study was undertaken. Contrary to our hypothesis, our evidence clearly showed no significant difference of Th9 cells and IL-9 mRNA throughout the development of the VMC. Data in cell frequencies and major related cytokine secretion levels failed to demonstrate Th9 cells could be differentially regulated in mice with VMC or not. There are at least two possible explanations for our observation. Firstly, as we known, IL-9 promoted the survival and activation of various cellular targets, including mast cells, B cells, T cells, and structural cells[27,28]. IL-9^+^CD4^+ ^cells were detected in all groups. So we hypothesis that Th9 cells might relate to both maintenance of cardic health and heart tissue destruction by CVB3. Secondly, although there are multiple cell types responsive to IL-9, and the biological effects of IL-9 are pleiotropic[29]. Many scholars believed that IL-9 was not obligatory for pathogenesis of some diseases and other cytokines can compensate its effect. For example, IL-9 had been shown to participate in the defense against helminth infections, asthma and allergy[17,30]. But some experiments demonstrated that IL-9 was not required for the expulsion of the intestinal parasitic nematode Nippostrongylus brasiliensis, the chronic allergen challenge induced bronchial mast cell accumulation, the development of allergen-induced pulmonary inflammation or airway hyperreactivity[31-33]. In our current experiment, no change of Th9 cells had been observed in VMC. From this point of view, we can't exclude the possibility that the pathogenic process of VMC is not associated with Th9 cells. Further investigation will be of particular interest to determine whether neutralize Th9 cytokines and/or in IL-9 mutant mice especially in IL-9/IL-9R-deficient mice model, lead to different viral immunity in the CBV3-induced VMC.

Th17 cells have been identified as a unique CD4^+ ^Th subset, which is characterized by production of pro-inflammatary cytokine IL-17[34]. Th17 subset has sparked great interest in the role of a broad range of immune-mediated tissue injury, including organ-specific autoimmunity in heart, VMC. Existing knowledge related to Th17 cells in VMC suggest that Th17 cells contribute to viral replication, facilitate the humoral immune response in acute VMC, and anti-IL-17 antibody can markedly reduce mice VMC severity[6-9]. Our experiment found Th17 cells highly expressed in the pathogenesis of VMC, which enriched from day 7, peaked on day 28, persisted at least to day 42 post-infection. But the change of Th9 cells did not reach statistical significance. It seems the microenvironments of VMC contribute to differentiation and proliferation of Th17 rather than Th9 cells. Meanwhile, although investigations in vitro had presented a complex regulatory network between Th17 and Th9 cells, and IL-9 could function as an autocrine growth factor that facilitates the expansion of Th17 cells populations[14,16,25,35,36]. It seems high expression of Th17 have no effect on Th9 cells in VMC. The exact relationship between Th17 and Th9 cells need future in vitro experiment to clarify.

There are several limitations in our study. Firstly, although the Th9 is the major CD4^+^T cell subset exciting IL-9, and we separately detected the CD4^+^IL-7^+^IL-9^- ^T and CD4^+^IL-9^+^IL-7^- ^T cells by flow cytometry, the present study cannot ascertain whether Th17 cells produced IL-9 in VMC[12,37]. Secondly, we focused on Th9 cells'major cytokine IL-9, but we did not extensively detect other cytokine such as IL-10, which is undetectable in human. Furthermore, we did not perform study in vitro and isolate Th17/Th9 cells to explore their relationship.

## Conclusion

Our preliminary data disclosed Th17 rather than Th9 cells significantly elevated in the development of CBV3 induced VMC mice, including cell frequencies and major related cytokine secretion levels. It seems the microenvironments of mice with VMC affect the differentiation and proliferation of Th17 but not Th9 cells. Whether Th9 cells participate in the pathogenesis of VMC warrants further research. The study of these manifestations might lead to new insight into the imbalance of Th17/Th9 cells in tissue inflammation and autoimmune responses, and the Th9 cells hypothesis might evolve into an even more intricate story.

## Competing interests

The authors declare that they have no competing interests.

## Authors' contributions

KQ coordinated the study, carried out data collection, performed the statistical analysis and interpretation of data, drafted the manuscript and reviewed it. WW participated in the conception and design, coordinated the study and reviewed it. YF, YY, PY and HY carried out data collection. All authors read and approved the final manuscript.
